# Effective Onboarding for Front‐Line Nurse Managers in Healthcare: A Descriptive Qualitative Study

**DOI:** 10.1155/jonm/9942076

**Published:** 2026-04-28

**Authors:** Markus Hakkarainen, Teresa Kunnari, Suvi Kuha, Tarja Hulkko, Outi Kanste

**Affiliations:** ^1^ Faculty of Medicine, Research Unit of Health Sciences and Technology, University of Oulu, Oulu, Finland, oulu.fi; ^2^ Wellbeing Services County of North Ostrobothnia, Oulu, Finland; ^3^ Medical Research Center Oulu, Oulu University Hospital, University of Oulu, Oulu, Finland, oulu.fi

**Keywords:** content analysis, front-line nurse manager, healthcare, interview, onboarding

## Abstract

**Aim:**

To describe nurse managers’ perceptions of effective onboarding for front‐line nurse managers in healthcare.

**Background:**

Many front‐line nurse managers are retiring, resulting in a loss of expertise. New competence requirements and widespread turnover intention among front‐line nurse managers are challenging healthcare organisations. Organisational support and effective onboarding are needed; however, qualitative research on the content of onboarding for front‐line nurse managers is lacking.

**Design:**

A qualitative descriptive study.

**Methods:**

In 2023, data were collected remotely through six semistructured group and five individual interviews with 18 nurse managers in Finland. The participants worked in specialist and emergency care, primary healthcare and medical services within a single healthcare region. The data were analysed using inductive content analysis.

**Results:**

The data yielded 33 subcategories and 12 categories that describe nurse managers’ views on effective onboarding for front‐line nurse managers in healthcare. The main categories were developing leadership growth through onboarding, clarifying leadership foundations in onboarding, designing a high‐standard and up‐to‐date onboarding process, ensuring well‐rounded onboarding support and strengthening workplace bonds through onboarding.

**Conclusions:**

Front‐line nurse manager onboarding requires systematic execution that includes personal, organisational and social aspects. Onboarding should focus on developing managers’ leadership qualities. Ensuring a structured and comprehensive onboarding programme that highlights the professional requirements of front‐line nurse managers, as well as the relevant theoretical and practical aspects of managerial work, can support a smooth transition into the new role. A primary limitation of this study is the potential influence of researcher subjectivity in data interpretation and analysis. To mitigate this, multiple researchers were involved in the analysis process to enhance credibility and reduce individual bias.

**Implications for Nursing Management:**

Individual onboarding should help new front‐line nurse managers grow into and develop attachments to the manager role while receiving support from top management, supervisors, colleagues and employees.

**Impact:**

This study highlights the need for a structured, personalised onboarding process for front‐line nurse managers, focussing on leadership development and multidimensional support. A well‐designed onboarding programme can enhance leadership skills, facilitate integration into the work community and support smoother transitions, ultimately contributing to improved organisational performance and healthcare outcomes.

## 1. Introduction

The outflow of healthcare professionals from the profession is constant, creating further pressure related to workforce competency and necessitating the development of strategies to ensure a competent workforce [1]. At the same time, many front‐line nurse managers are retiring, resulting in a substantial loss of expertise in nursing leadership [2]. The widespread intention to leave among front‐line nurse managers further challenges healthcare organisations globally [3, 4], with over 50% considering leaving their positions within the next 5 years [5].

To mitigate turnover among front‐line nurse managers, healthcare organisations should implement comprehensive organisational strategies, including orientation, coaching, mentorship, leadership development programmes, opportunities for career progression and stress management interventions [3, 4]. Along with factors affecting work well‐being, job attractiveness should also be considered, as problems in healthcare generally relate to unclear employer branding, non‐pecuniary job characteristics, contract durations, wages and healthcare sectors [6, 7].

Front‐line nurse managers occupy a critical position within healthcare organisations, serving as the primary leaders responsible for coordinating daily unit functions and supervising the delivery of nursing services [8]. The work of front‐line nurse managers encompasses a broad scope of responsibilities, including staffing, budgeting, care coordination and policy compliance [9]. The complexity of this position requires learning new management skills in constantly changing healthcare organisations [10]. However, the current level of competence among front‐line nurse managers is often inadequate. A significant proportion of front‐line nurse managers enter these roles without prior leadership experience or formal educational preparation. Without educational prerequisites and organised competency development initiatives, such as onboarding, knowledge acquisition tends to occur through informal, experiential learning. While practical experience is valuable, reliance on trial‐and‐error learning is insufficient and inefficient when developing a vital workforce [2]. Therefore, creating a targeted and structured onboarding process for front‐line nurse managers is essential.

## 2. Background

The concept of onboarding first appeared in academic literature in the 1970s, primarily through the work of Van Maanen and Schein [11], who introduced key terms such as organisational socialisation, newcomers, insiders and outsiders. A widely cited definition by Bauer and Erdogan [12] equates onboarding with organisational socialisation, describing it as the process through which new employees transition from being external entrants to fully integrated members of the organisation. However, more recent perspectives view onboarding as a broader concept that encompasses both orientation activities and socialisation processes [13].

Onboarding can be viewed through the lens of adult learning theories, particularly social constructivism, which emphasises that knowledge is coconstructed through interpersonal interactions. This theoretical framework suggests that learning is inherently a social endeavour [14]. The primary objective of onboarding is to equip new hires [13] with the essential information and competencies necessary for effective performance within the organisation. It is a critical component of the recruitment process, acting as a systematic approach to facilitating the integration of new employees into the organisation’s culture and operational framework [14]. Many benefits associated with onboarding have been identified, such as increased organisational commitment, employee engagement and retention, organisational success and improvement in new employees’ experience [15].

During orientation, a seasoned employee facilitates the integration of a new employee by providing support, training and guidance to help them acclimate to the organisational environment and culture [16]. Front‐line nurse managers have reported negative orientation experiences due to limitations and poor quality [17]. Some front‐line nurse managers are unprepared for their roles upon starting a new position [17–19], leaving them to learn essential skills independently through engagement in everyday work without formal orientation [17]. The transition to front‐line management is an experimental journey shaped by role ambiguity, loneliness and unpreparedness [20]. This leap into a managerial position can cause emotional stress [17], confusion and a sense of constant need for support, as well as ineffectiveness at work [18, 19]. Elevated stress levels resulting from insufficient role‐specific knowledge are a primary contributor to burnout and turnover among front‐line nurse managers. The absence of a systematic orientation programme has been linked to increased turnover rates, reduced patient satisfaction, compromised succession planning and diminished quality of care outcomes [10].

Socialisation is a core component of onboarding, as it enables new hires to feel connected to the organisation and to develop relationships within the community [13]. It is an inevitable [21] and dynamic process involving learning, interaction, development and adaptation. Professional socialisation is shaped by training opportunities, role models, organisational support structures, practical experience and feedback [22, 23]. Successful onboarding, therefore, requires building trust and enabling meaningful engagement with colleagues, which supports learning about others and familiarising oneself with the broader organisational context [13].

Mentoring and coaching are also important components of the onboarding process [13, 24]. Mentoring encompasses functions such as coaching and teaching and serves as a mechanism for cultivating professional knowledge, competencies and values and for assimilating into organisational culture [25]. Coaching, in turn, refers to a structured developmental interaction that employs appropriate strategies, tools and techniques to strengthen the coachee’s skills and performance [26]. Guidance, including vocational guidance, involves supporting individuals in making informed decisions about their professional career paths and taking appropriate action [27].

A structured and comprehensive onboarding programme can be effective in increasing engagement among front‐line nurse managers. Formalised onboarding programmes for front‐line nurse managers focused on structured mentorship [20], coaching, role preparation and transformational leadership practices can enhance front‐line nurse managers’ engagement and retention. The transition to the role of front‐line nurse manager can be supported with various tools, such as an eManual, to guide the onboarding process and facilitate leadership development. [24, 28]. Shortcomings have been identified in the orientation and training of managers regarding the use of applications, software and information and communications technology tools for employees. It is essential to provide front‐line nurse managers with precise and relevant education, along with ongoing mentorship and support, to enable them to utilise emerging technologies effectively [20, 29].

Key findings from previous research indicate that successful onboarding of front‐line nurse managers requires support from organisational leadership, organisational practices that facilitate orientation and an orientation period that includes didactic coaching, with continuous content evaluation [19]. However, in evaluating the learning needs of front‐line nurse managers, researchers have identified a scarcity of resources designed to support the transition to the front‐line nurse manager role and leadership growth [20, 24]. Transition‐to‐practice programmes [30] and onboarding programmes [19, 24] have a positive impact on professional competence and role transition; however, front‐line nurse managers continue to experience challenges related to the support provided during their transition to managerial roles. In addition to these organisational shortcomings, some individual coping mechanisms also shape how front‐line nurse managers manage the transition [20]. Overall, despite increasing recognition of the importance of structured onboarding, a limited amount of qualitative research has focused on the content and components of effective onboarding for front‐line nurse managers. This general lack of qualitative evidence underscores the importance of developing context‐sensitive onboarding programmes that can be adapted across diverse healthcare systems.

Hence, this study aimed to describe nurse managers’ perceptions of effective onboarding for front‐line nurse managers in healthcare.

## 3. Materials and Methods

### 3.1. Research Questions

The research question was as follows: What are nurse managers’ perceptions regarding effective onboarding for front‐line nurse managers in healthcare?

### 3.2. Study Design

A qualitative, descriptive study design was employed to explore a phenomenon that remains incompletely understood [31]. This design allowed participants’ perceptions to be described in real‐life situations [32]. This study followed the Consolidated Criteria for Reporting Qualitative Research (COREQ) guidelines.

### 3.3. Study Setting and Recruitment

The data were collected from nurse managers employed in specialist and emergency care, as well as primary health and medical services, in one Finnish healthcare region in 2023. The region employs over 18,000 healthcare professionals across various fields, with a population of approximately 418,000.

Purposive sampling was used to recruit participants [31]. The inclusion criteria for this study encompassed all nurse managers currently working in the target organisation, regardless of their professional experience or tenure in a managerial position. Exclusion criteria included top‐level managers, such as directors or executives. Interview invitations and background information related to the study were emailed to contact persons at various organisations, who then forwarded them to potential participants. A total of 49 nurse managers were identified, and invitations were sent; 18 interviewees participated in the study. Nine were invited but declined due to time constraints, and 22 could not be reached despite several attempts. The background information was collected using an electronic Webropol survey. Sixteen participants were employed in specialised care, and two worked in primary care. Twelve participants were front‐line nurse managers, while the others held middle management positions (Table 1).

**TABLE 1:** Nurse managers’ demographics (*n* = 18).

Variable		*f*
Gender	Female	16
	Male	2

Educational level	Master’s degree or higher	11
	Bachelor’s degree or lower	7

Organisation	Primary healthcare	2
	Specialised care	16

Work task (position)	Front‐line nurse manager	12
	Middle management position	6

Age		Mean 52.7 (SD: 7.7) range: 37–64 years

Work experience	In the current work	Mean 2.2 (SD: 3.8) range: 1 month–15 years
	In healthcare management positions	Mean 12.8 (SD: 7.4) range: 2 months–27 years

Abbreviation: SD, standard deviation.

### 3.4. Data Collection

The data were collected through semistructured group interviews (*n* = 6) with two to three participants per group and individual interviews (*n* = 5). Group interviews were chosen as the data collection method since the setting allows interviewees to exchange ideas, giving a broader perspective [31]. Some participants were unable to attend the planned group interviews due to scheduling constraints and other unforeseen absences; therefore, they were interviewed individually.

The interviews were conducted by research group members (*n* = 2) and research assistants (*n* = 5), who were prepared to follow standard guidelines while conducting the interviews. One or two interviewers participated in the interviews. Seven interviewers (three men and four women) were Master of Health Sciences students with a healthcare professional background. The interviewer and the interviewees did not know each other in advance. The interviews were conducted between March and December 2023 via Microsoft Teams video calls during participants’ work hours and at a time that suited their schedules. No one else was present, except for the participants and researchers, during the interviews. The interviews were recorded with the participants’ permission. Notes were also made during the interviews. The interview guide was sent to the participants beforehand. At the end of each interview, participants had the opportunity to add comments on the interview topics.

The semistructured interview guide used was based on previous research [13, 19, 33] focussing on onboarding for front‐line nurse managers. The interview guide was developed by the research group, which included researchers with experience in conducting qualitative research on nursing management (Table 2). The interview guide was pretested with two people working as nurse managers in a hospital organisation. Based on pretesting, the number of questions was reduced, and the wording of some questions was clarified. The pretesting interviews were not included in the sample of this study.

**TABLE 2:** Semi‐structured interview questions.

Interview questions
What methods would support the onboarding of front‐line nurse managers?
What competencies should front‐line nurse managers achieve during onboarding?
How could mentoring be utilised during onboarding for front‐line nurse managers?
What support do front‐line nurse managers need from the organisation during the onboarding process?
What kind of support do front‐line nurse managers need from the work community during the onboarding process?
What can be done to promote front‐line nurse managers’ attachment to the work community and culture?

The duration of the focus group interviews ranged from 51 to 68 min, and individual interviews ranged from 39 to 57 min (total duration: 618 min). The researchers discussed the content of the data as the interviews progressed and agreed that it became saturated after 15 interviews.

### 3.5. Data Analysis

Inductive content analysis was employed for data analysis, as there was no comprehensive previous research on the phenomenon under investigation, and human experiences were of interest [32]. The recorded interviews were transcribed. The interview transcripts consisted of 85 pages, each in 11‐point Calibri (body) font with a line spacing of 1.

All the collected data from the group and individual interviews were combined and analysed inductively, and categories were identified through this analysis. Firstly, two researchers (blinded) went through the data separately, looking for original expressions related to the research question. Individual meanings were chosen as the unit of analysis because the study aimed to investigate concrete experiential topics [32]. After identifying the original expressions, they were simplified to convert them into open codes (*n* = 514) using Microsoft Excel software. Then, the open codes were grouped into sub‐categories (*n* = 33), categories (*n* = 12) and main categories (*n* = 5) based on similar meanings, which were given descriptive names (Supporting Figure 1). Boundaries between categories were ensured by emphasising the authentic content of original expressions while simultaneously focussing on avoiding overlap between categories. The researchers (blinded) had regular meetings with the research group (blinded) during data collection and analysis and shared results throughout the analysis. Any disagreements or differing interpretations were systematically addressed through collaborative discussions to ensure consistency and consensus in the analysis.

### 3.6. Ethical Considerations

Before the interviews, participants received written and verbal information about the study and the researchers, as well as a data protection notification. Participation was voluntary, and informed consent was ensured by providing participants with written information about the study purpose, procedures, confidentiality and their right to withdraw at any point without consequences for their employment. Informed consent was obtained both verbally and electronically before the interviews [34].

In Finland, research involving human participants is guided by the ethical principles of the Finnish National Board on Research Integrity (TENK), which the University of Oulu adheres to. This study was not reviewed by a Finnish human sciences ethics committee, as it did not include any of the review‐triggering elements specified in TENK guidance. Specifically, the research did not deviate from informed consent procedures, did not involve interventions affecting participants’ physical integrity, did not include minors without appropriate consent, did not expose participants to exceptionally strong stimuli, did not pose a risk of mental harm beyond that encountered in everyday life, and did not entail threats to the safety of either participants or researchers. On this basis, an ethics committee statement was not required [35]. Organisational permission was obtained solely to allow data collection within the workplace and did not involve access to identifiable employee information. This organisational permission is distinct from ethical review. A separate description of the Finnish ethical review system was independently assessed by the Ethics Committee of Human Sciences at the University of Oulu (Supporting File 1).

The researchers and participants were not previously acquainted. All personal data were removed during the transcription of the interviews to ensure the participants’ anonymity. The security and integrity of the data were protected, including secure storage and appropriate data access restrictions in accordance with the General Data Protection Regulation (GDPR) [36].

### 3.7. Rigour

The trustworthiness of this qualitative study is based on its credibility, transferability, dependability, confirmability and authenticity [32, 37]. To enhance the study’s credibility, interviewees were selected on a discretionary basis using a purposive sampling strategy. Data saturation was achieved during the interviews. To improve the transferability of the results, the interviewees’ background information is described. However, using a single Finnish region may limit the transferability of the findings to differing organisational structures and to international contexts. Researcher triangulation was employed to enhance the credibility and confirmability of the findings by involving multiple researchers in the data analysis process. Two researchers analysed the data, and two others cross‐checked the final analysis.

Reflexivity was maintained throughout the study, and researcher triangulation helped mitigate potential researcher bias. The researchers systematically reflected on their own positionalities and subjective interpretations during the data analysis. Sharing the same cultural context and professional experience within the healthcare system as the informants, researchers acknowledge that this positioning influences the interpretation of findings. One researcher held a similar managerial position as the participants. This person did not participate in coding, and interpretations were reviewed within the research group; the entire analysis process was documented transparently. The confirmability of the research is enhanced by including a transparent description of the research process, thereby making interpretations traceable. Using interview citations when reporting results contributes to the study’s authenticity [32].

## 4. Results

**FIGURE 1:**
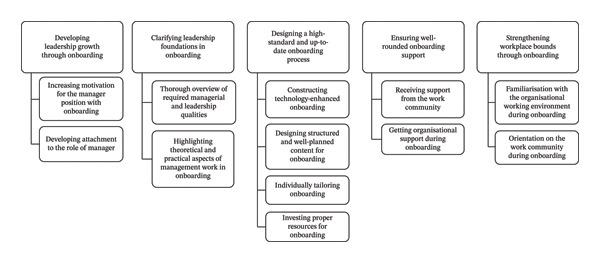
Overview of effective onboarding for front‐line nurse managers in healthcare.

Analysis of the data revealed four main categories in nurse managers’ views of the effective onboarding of front‐line nurse managers: (1) developing leadership growth through onboarding; (2) clarifying leadership foundations in onboarding; (3) designing a high‐standard and up‐to‐date onboarding process; (4) ensuring well‐rounded onboarding support; and (5) strengthening workplace bonds through onboarding. Figure 1 presents the five main categories and the categories under each, generated through inductive content analysis.

### 4.1. Developing Leadership Growth Through Onboarding

The first main category included the following two categories: increasing motivation for the manager position with onboarding and developing attachment to the role of manager.

Based on the nurse managers’ views, *increasing motivation for the manager position with onboarding* can be achieved. Nurse managers mentioned that high‐quality and systematic onboarding increased the attractiveness of the front‐line nurse manager position. Participants noted that increasing front‐line nurse managers’ commitment to their work can be achieved during onboarding, for example, by offering job counselling and having regular meetings with colleagues and supervisors.

‘… It does increase your commitment to the work, in that you get training, and you get a proper onboarding process.’ Nurse manager/Individual interview 1

‘… And also, job counselling as well as the chance to develop during the onboarding increases role commitment.’ Nurse manager/Individual interview 3

According to nurse managers, developing an attachment to the role of manager can be facilitated in various ways during the onboarding process. Growing into the managerial role can be supported by clearly outlining the expectations of the role and ensuring that the front‐line nurse manager’s supervisor provides adequate support. Job counselling, coaching, mentoring and finding one’s strengths and weaknesses were likewise seen as means of growing into the manager’s role. Nurse managers noted that transitioning from a nurse to a front‐line nurse manager requires familiarity with the role of a manager. The participants expressed that the front‐line nurse manager’s job description and responsibilities should be clearly and comprehensively highlighted before and during onboarding. It was seen as essential to highlight processes that the front‐line nurse manager will be responsible for.

‘… When you are beginning as a [front‐line] nurse manager, you need some sort of job counselling and guidance to support you as you grow into the new role.’ Nurse manager/Group interview 3

‘… It would be invaluable for the contents of the manager’s work to be outlined and themed.’ Nurse manager/Group interview 2

### 4.2. Clarifying Leadership Foundations in Onboarding

The second main category included the following two categories: a thorough overview of the required managerial and leadership qualities and highlighting the theoretical and practical aspects of management work in onboarding.

Providing a *thorough overview of the required managerial and leadership qualities* was considered an essential part of the onboarding process. According to participants, enhancing competence for daily managerial work should be included in onboarding. This was seen to include competence regarding economics, risk management, guidance skills, skills involving personnel, student‐related matters and procedures for applying for further education. Ensuring competence in service chain management was also mentioned by the participants, including know‐how related to assessing services, analysing patient demographics, patient safety and feedback. According to the participants, self‐management and leadership include the management of one’s time, setting boundaries between work and free time, detachment from work and recovery, self‐awareness as a manager and prioritising received information. Support from the person giving the orientation and general support regarding time‐related pressure were seen as essential factors regarding self‐management and self‐leadership.

‘And well, negotiation skills, guidance skills, participatory skills, and meeting‐related skills, at least, that’s what I can think of.’ Nurse manager/Group interview 5

‘Means of assessing the services received by patients should be included.’ Nurse manager/Individual interview 4

‘…The orientation should emphasise that… you have to push work back and create boundaries between your work and free time.’ Nurse manager/Group interview 3

Nurse managers felt that *highlighting the theoretical and practical aspects of management work in onboarding* is necessary. This included management practices and theories such as change management, management by coaching, motivational management, competence management, knowledge management and other topical management practices. According to the participants, orientation related to human resources management practices should comprise shift planning and procedures regarding absent personnel and sick leave documentation. Additionally, the management of work well‐being, knowledge management for personnel and human resources management in general were also considered essential.

‘In particular, current knowledge on management practices should be included.’ Nurse manager/Individual interview 3

‘Broadly speaking, we need a lot of human resources management knowledge in this position.’ Nurse manager/Group interview 5

### 4.3. Designing a High‐Standard and Up‐to‐Date Onboarding Process

The third main category included the following four categories: constructing technology‐enhanced onboarding, designing structured and well‐planned content for onboarding, individually tailoring onboarding and investing proper resources for onboarding.

According to nurse managers, *constructing technology-enhanced onboarding* should be diverse and thorough. Orientation on technological systems was seen as an essential part of front‐line nurse manager onboarding. This included listing all the systems used by front‐line nurse managers in their daily work, and then giving a comprehensive orientation on each. Furthermore, orientation on systems used to gather information and create reports, as well as distance working tools, was seen as important. Common challenges relating to new technological tools were the difficulty in finding orientation material and instructions regarding the use of technological tools. Also, errors in systems were found to make learning technological tools more difficult. Based on participants’ views, the use of clear digital orientation materials supports onboarding. A digital orientation course that includes all the essential information and guidelines for front‐line nurse managers was seen to be useful. Functional tools and environments, such as working user identifiers for computer systems and easy access tools supporting work, were seen as a key supporting factor in the onboarding process.

‘Well, of course, technological matters, considering gathering information and creating reports were included in my orientation.’ Nurse manager/Individual interview 4

‘Overall, considering technology and these systems in use, they are not integrated well enough. So all of the systems should be integrated.’ Nurse manager/Group interview 1

‘A Clear online course regarding certain practices would support the onboarding.’/Nurse manager/Group interview 3

‘The working environment and the tools used in the work should be in order, so that they support orientation to the new position.’ Nurse manager/Group interview 2

According to participants, *designing structured and well-planned content for onboarding* can be achieved by using up‐to‐date orientation material to support the onboarding and by ensuring an organised onboarding structure. Up‐to‐date materials provide support for decision‐making in daily operations, and they are seen as especially important when the front‐line nurse manager comes from outside the organisation. Also, information and materials should be passed on by the predecessor, including a clear list of key contacts for day‐to‐day work. Based on these views, a dedicated person should be responsible for updating the necessary materials. According to the participants, different forms should be created alongside the checklists to support the onboarding by providing structure. Structurality was seen to prevent too much information from being communicated at once and to provide a clear format for onboarding. Experiences on shortcomings in the implementation of systematic onboarding varied. Some participants said that there was no onboarding to begin with or that there was no unified plan behind onboarding for front‐line nurse managers. The majority of the identified shortcomings were related to inadequate onboarding as a whole.

‘Especially when a manager comes from outside the organisation, onboarding materials should be up to date.’ Nurse manager/Individual interview 2

‘Informational orientation should follow a structured model with checklists, etc. It should be available for viewing before the actual employment begins and after the onboarding.’ Nurse manager/Group interview 4

Based on participants’ views, adequate onboarding requires proactivity from the front‐line nurse manager, and onboarding should be customised to the needs of the individual. Together, these factors were seen to support *individually tailoring onboarding*. Proactivity on the part of front‐line nurse managers involves independently seeking out education on various topics and self‐orientation on different areas of managerial work. According to the participants, onboarding should be customised based on the background and prior experiences of the front‐line nurse manager. For example, the onboarding should focus on human resources management or daily work, depending on the individual’s previous experience.

‘It is the manager’s own responsibility to discover where they can find education.’ Nurse manager/Individual interview 1

‘The contents of the onboarding should be customised to the new manager’s background.’ Nurse manager/Group interview 4

Investing proper resources for onboarding was seen as important for proper onboarding to take place. Based on nurse managers’ views, adequate time for onboarding should be ensured by the organisation, and this was seen to increase dedication to managerial work. Additionally, having sufficient time for conversations with personnel and one’s supervisor was considered significant. Having enough time was seen to improve learning during onboarding. An important challenge was the limited time allocated for onboarding. This included insufficient time for the process as a whole, as well as opportunities for onboarding‐related discussions, for which time was not allocated. The nurse managers raised the need to appoint a support person to coordinate the onboarding. A support person is seen as someone who gives guidance related to daily work. Also, it was expected that the front‐line nurse manager could ask the support person for help when needed. Sometimes, no coordinator was involved in the onboarding process. It was also considered essential to receive information and work‐related documents from one’s predecessor; however, these were sometimes unavailable. According to the participants, involving specialists in interdisciplinary onboarding meant having specialists in certain tasks teach sections of their expertise as part of the onboarding process, i.e., nurses contributing to orientation on clinical work and information technology staff guiding on information technology‐related issues.

‘The onboarding should be scheduled for a longer period of time, because the amount of information that you can internalise at any one time is limited.’ Nurse manager/Individual interview 4

‘Well, maybe it’s best to name a particular person to be your security support. And best of all would be that person takes you under his or her wing and takes the time needed for it [onboarding].’ Nurse manager/Individual interview 2

‘Well, along with colleagues and one’s own superiors, the clinical secretary. They would give us guidance on how to operate and give information.’ Nurse manager/Individual interview 5

### 4.4. Ensuring Well‐Rounded Onboarding Support

The fourth main category included the following two categories: receiving support from the work community and getting organisational support during onboarding.

According to the nurse managers, *receiving support from the work community* during the onboarding process is essential. Participants sought versatile assistance from colleagues during the onboarding process. Colleague support was valued in terms of resolving challenging situations, having regular meetings to handle stress and utilising peer learning. Nurse managers also highlighted interactional help from staff during onboarding. Participants hoped for good communication and an open atmosphere between staff and the front‐line manager. Additionally, support and understanding from staff during the onboarding process were desired. According to the nurse managers, support from the work community during onboarding was sometimes lacking, which posed challenges during the onboarding process. Based on participants’ views, staff often have justified expectations of a front‐line nurse manager, but responding immediately is usually challenging. During the onboarding process, the front‐line manager’s supervisor was expected to provide regular guidance, particularly on decision‐making and leadership principles. Nurse managers also believed that mentoring is an effective way to support onboarding, particularly for individuals with no prior management experience. According to the nurse managers, communication with mentors can be more frequent in the early stages of onboarding and become less frequent in later stages.

‘I experience it so that the good work atmosphere and a friendly and supportive way of work, I think it is quite good.’ Nurse manager/Individual interview 4

‘When you become a new [front‐line] nurse manager in a work community, a kind of open interaction [from staff] … would keep the new nurse manager up to date.’ Nurse manager/Individual interview 3

‘When the [front‐line] nurse manager changes, it is often a kind of crisis for the work community and the staff, so the support for the [front‐line] nurse manager may be forgotten by some.’ Nurse manager/Individual interview 2

‘Of course, there is also the hope that the [front‐line] nurse manager’s supervisor would reach out with a listening ear for the new [front‐line] nurse manager or the [front‐line] nurse manager working in a new unit, recognising that the [front‐line] nurse manager also requires support, and inquiring how he/she is doing.’ Nurse manager/Individual interview 4

‘I think mentoring is important.’ Nurse manager/Individual interview 5

Participants considered *getting organisational support during onboarding* to be an essential part of the onboarding process. The importance of front‐line nurse managers receiving comprehensive organisational support was emphasised. According to the participants, clear and consistent practices within the organisation are needed to support onboarding. For example, participants felt that clear procedures and strategic guidelines within the organisation support onboarding. Nurse managers suggested that the organisation can also support onboarding by providing regular leadership training for front‐line nurse managers. According to the participants, a lack of organisational support can make the onboarding process more challenging. Participants reported that reducing support structures made the onboarding process more complicated. Some nurse managers felt that not enough attention is paid to the training of front‐line nurse managers and that support from higher levels of the organisation was often insufficient. It was also considered important that organisational support services, such as the human resources department and information and communications technology department, assist during the onboarding process. Participants wanted it to be more straightforward to reach out to these support services.

‘Perhaps the first thing that comes to my mind about organisational support is clear practices and common guidelines’ Nurse manager/Individual interview 2

‘And if you could work with them [human resource services] to figure out specifically what the new manager should know about their input in a work unit.’ Nurse manager/Group interview 2

### 4.5. Strengthening Workplace Bonds Through Onboarding

The fifth main category included the following two categories: familiarisation with the organisational working environment during onboarding and orientation on the work community during onboarding.

The participants found that *familiarisation with the organisational working environment during onboarding* was essential. Nurse managers highlighted that gaining insights into the organisation and its guiding principles during onboarding is essential. Orientation related to the particular work unit and its policies was seen as important; for example, participants highlighted that information on the operations, practices and processes of the work unit should be communicated. It was also considered important to get to know the work of nurses by being present in the unit and to get to know the needs of the people who use the services of the unit. According to the nurse managers, networking with partners within the organisation is an important part of the onboarding process. Nurse managers mentioned that networking with partners and getting to know their operations is essential.

‘When you come from outside the organisation, there should be orientation on the organisation’s values and operations.’ Nurse manager/Group interview 6

‘An introduction to the organisation, going through what the organisation is and how the responsibilities and tasks are divided, and introducing how we do things here.’ Nurse manager/Individual interview 5

‘I think that networking with the people working around you is very important as well.’ Nurse manager/Group interview 2

The participants’ responses indicated that *orientation on the work community during onboarding* was an essential part of onboarding. Gaining trust with the new work community was seen as crucial to socialisation in the work community. It could involve taking care of daily work and hearing out about the experiences of personnel. Also, trusting communication between the front‐line nurse manager and personnel was seen as a supporting factor. According to the participants, the introduction to the work community should occur during the front‐line nurse manager’s first days, through brief conversations. Also, it was seen as supportive when the front‐line nurse manager’s supervisor introduced them to the work community. Participants considered that familiarisation with the work community during the onboarding process was essential. Familiarisation with the work community was achieved through interaction and communication with personnel, as well as by building relationships within the work community. Being present in the work community, listening to staff and introducing oneself were seen as supporting factors. In addition, the front‐line nurse manager’s interpersonal skills and getting to know the culture were seen to affect socialisation.

‘My experience is that you take care of things and that you need to show the staff that you are taking their concerns into account.’ Nurse manager/Group interview 6

‘On the first day of work, it would be good if there was someone to introduce the new [front‐line] nurse manager, if it is a new work unit.’ Nurse manager/Group interview 1

‘And the fact that you kind of build that trust with staff and also with future colleagues, the other [front‐line] nurse managers.’ Nurse manager/Group interview 5

## 5. Discussion

This qualitative study aimed to describe nurse managers’ perceptions of effective onboarding for front‐line nurse managers in healthcare. The results show that developing leadership growth through onboarding can help increase motivation and commitment to the management position. Previous studies have also found that front‐line nurse manager engagement can be effectively improved with a structured and comprehensive onboarding programme [24, 28]. The results of our study align with previous studies [20], suggesting that developing an attachment to the front‐line nurse manager role is a crucial aspect of onboarding. Previous studies have also found that attachment to the manager role can be achieved by creating relationships and learning skills in a new professional role, where new employees receive support from more experienced employees during orientation as they adapt to a new work environment and culture [13, 16]. Similar support can be enhanced through coaching, which can help align professional development with relevant career trajectories [26, 27].

Our results also highlighted that clarifying leadership foundations required for front‐line nurse manager work is an essential part of the onboarding process. Ensuring that new front‐line nurse managers receive a structured overview of the managerial competencies and leadership expectations associated with the role is important, as a lack of knowledge in these areas increases stress and turnover among front‐line nurse managers [10]. Our results emphasise the need to address both theoretical and practical aspects of managerial work during onboarding. A previous study demonstrated that a comprehensive orientation programme covering financial, human resource, strategic and performance management significantly improved managerial competence across all domains [10].

We found that designing a high‐standard and up‐to‐date onboarding process, such as constructing technology‐enhanced onboarding, was seen as an essential part of the onboarding process. For example, previous studies have demonstrated that an eManual can effectively guide the onboarding process [24, 28]. However, based on our results, utilising technology also presents challenges for front‐line nurse managers during onboarding, such as functional problems with many technological tools. Sharpp et al. [29] reported similar challenges related to technology use during onboarding, while Esquisábel‐Soteras [20] also highlighted the need for relevant education in the use of information and communications technology tools. However, remote work is becoming increasingly common, referring to the shift from in‐person to remote onboarding. This involves utilising digital tools and technologies to facilitate onboarding, including video conferencing, virtual workplace tours and online training modules [15].

Our results further highlight the importance of structured, well‐planned onboarding content, including up‐to‐date materials and clear structural elements. Shortcomings in the implementation of systematic onboarding were also noted. This may reflect inconsistencies in onboarding practices, a lack of standardised procedures or limited organisational investment in onboarding design. As Coogan and Hampton [10] found, the lack of systematic orientation programmes has diverse effects on front‐line nurse managers and their working environments. Accordingly, when onboarding is implemented as a structured and comprehensive process, it can strengthen engagement, support retention and reduce turnover among front‐line nurse managers [3, 24, 28]. Consequently, our results align with previous studies that have found front‐line nurse manager onboarding to be limited and of poor quality, resulting in learning through trial and error, which is neither sufficient nor efficient [2, 10, 17, 18].

The results of our study highlight the need for individualised onboarding tailored to each person’s skills and emphasise that front‐line nurse managers need to be proactive during onboarding. Previous studies have noted the value of transformational leadership practices in onboarding [24, 28]; however, prior research has not explicitly identified managerial proactivity as a central prerequisite for onboarding. Our study, therefore, extends existing knowledge by demonstrating that front‐line nurse managers need to take a proactive role to receive comprehensive onboarding, thereby placing part of the responsibility on the front‐line nurse managers themselves. This previously underexplored perspective may indicate organisational expectations of managerial autonomy or reflect gaps in structured onboarding processes, thus offering a novel contribution to the literature.

Our results highlight the need to invest sufficient resources in onboarding, including adequate time. According to Carlos and Muralles [13], adequate time is crucial since meeting other colleagues and understanding ideas and relationships require sufficient and intentionally allocated time. According to our study, a dedicated person should be responsible for the onboarding. Ward and McComb [16] also found that a more experienced employee can support, train and help a new employee to adapt to a new work environment and culture during orientation. According to our results, onboarding without a designated coordinator was perceived as challenging. This may be due to a lack of structured guidance, unclear responsibilities and inconsistent support during the onboarding process. Such challenges can lead to uncertainty in role expectations, slower adaptation and potentially reduced managerial confidence in the early stages of the position. Previous studies also found that front‐line nurse manager onboarding is more challenging when no one person provides the orientation or there is no assigned mentor [16, 17].

Based on our results, ensuring well‐rounded onboarding support for front‐line managers is essential. We found that receiving support from the work community during onboarding is part of a multidimensional support system. Previous studies have not identified such a diverse range of support sources; however, the value of peer support has been previously noted [20]. These findings suggest that onboarding is simultaneously an organisational, managerial and socially embedded process rather than a set of isolated support activities. Carlos and Muralles [13] found that best practices for onboarding include seeking regular feedback from colleagues and holding regular meetings with them, ideally with a loose agenda, which provides opportunities to brainstorm, process, collaborate and check in with one another. Our results suggest that the front‐line nurse manager’s supervisor is expected to provide regular guidance during the onboarding process. This ongoing support appears essential for building managerial confidence, clarifying role expectations and ensuring a smoother transition to the new position. Warshawsky et al. [19] found that new front‐line nurse managers believe it is imperative that their onboarding be viewed as necessary by all leaders in the organisation. Previous research supports our finding that mentoring is an essential part of the onboarding process [13, 19, 20, 24, 25]. Our findings highlight that mentoring is especially valuable for individuals lacking prior management experience, despite Warshawsky et al.’s [19] assertion that mentorship should be standard practice for everyone.

The results of this study show that getting organisational support during onboarding is essential. Nurse managers hoped that organisational guidelines would be included in the onboarding process. Earlier studies support our finding that successful onboarding requires the organisation to implement practices to support the onboarding process [19, 30]. A previous study also demonstrated that a successful transition to the front‐line nurse manager role begins with organisational policies that support the development of managerial competence [19]. We found that onboarding for front‐line nurse managers and the onboarding‐related support received from the organisation were often inadequate; some previous studies have reported similar findings [17–19]. The lack of organisational support is clearly detrimental to the implementation of a successful onboarding process [19, 30].

Our results indicate that onboarding should actively facilitate social integration by enabling familiarisation with the organisational working environment and the work community, thereby strengthening workplace bonds. This is important, as previous studies have noted that the transition to front‐line management is characterised by significant role uncertainties and loneliness [20]. Strong social integration has been shown to enhance role clarity, psychological safety and the long‐term retention of new front‐line nurse managers. Similarly, previous studies emphasise that professional socialisation, through interaction with and adaptation to the work community, is central to the development of a professional identity [21–23]. Based on our results, gaining insights into the organisation, orientation on the work unit and interaction with partners are part of this familiarisation with the working environment. To promote this, it has been suggested that a transition‐to‐practice programme is an effective means of learning policies and resources specific to the front‐line nurse manager’s organisation [30].

According to our results, gaining trust in the new work community is crucial for successful orientation within it. Carlos and Muralles [13] similarly found that it is possible to establish trust between the new hire and other staff through the onboarding process. This is particularly relevant, given that new front‐line nurse managers often report insufficient support during their transition to managerial roles [20]. We further found that familiarisation with the work community during onboarding facilitates deeper relationship‐building and strengthens commitment to the workplace. A previous study found that providing meaningful time for new hires to learn about the community enables them to develop a deeper connection to their work. This deep investment in relationship‐building and trust‐building among individuals is a crucial element in the successful onboarding of new hires. [13].

### 5.1. Limitations

This study has some limitations. The data were collected from a single healthcare region in a single country, which may limit the transferability of the results to other organisations or countries. However, the results can be applied in different contexts to develop effective onboarding practices. The interview guide was pretested with two people to confirm its adequacy. Seven interviewers participated in data collection, two researchers were involved in data analysis, and the results were verified with two other researchers. One potential limitation of this study is social desirability bias, as participants may have shaped their responses to align with perceived social norms or expectations. Conducting interviews remotely may also have limited the depth of interaction and contextual sensitivity, potentially affecting the richness of the data.

One limitation is the potential influence of researcher subjectivity, given that reflexivity plays a central role in data interpretation and analysis. To mitigate this, multiple researchers were involved in the analysis process to enhance credibility and reduce individual bias.

Researcher triangulation was applied; however, methodological triangulation and member checking were not implemented due to limited resources and time constraints. These may affect credibility and are explicitly recommended for future studies to strengthen trustworthiness. The absence of these may undermine trustworthiness, as interpretations rely primarily on researchers’ perspectives and lack participant validation or comparison across multiple methods. The researchers may have had different preconceptions about the phenomenon, which could have influenced data collection and analysis. However, all researchers shared standard interview guidelines. The transcripts were not returned to participants for their comment or confirmation of authenticity. However, during the interviews, all participants confirmed their statements by stating that they had nothing further to add, suggesting they had fully disclosed the matter. Additionally, one researcher held a managerial role similar to that of the informants, and this was taken into account during the validation of the findings.

### 5.2. Recommendations for Further Research

Further research should focus on the transition of nursing and healthcare professionals from clinical to managerial roles, as this represents a significant challenge in preparing and effectively managing healthcare systems. Developing a comprehensive, up‐to‐date onboarding programme that effectively utilises digital technologies and artificial intelligence, and assessing its effectiveness through an intervention study, is essential. Additionally, using data and methodological triangulation, as well as subgroup analyses, is recommended to enhance the robustness and transferability of the findings. Including middle managers in the study may have affected the findings, as their perspectives may differ from those of front‐line managers regarding responsibilities, decision‐making and onboarding experiences. Therefore, it would be necessary to investigate these subgroups separately in future studies.

### 5.3. Implications for Nursing Management

Our study highlights the need for meaningful, systematically structured onboarding for front‐line nurse managers. Individual onboarding based on professional knowledge and skills should facilitate growth and attachment to the front‐line nurse manager role. Consequently, onboarding should aim to embrace front‐line nurse managers’ prior knowledge and address their professional weaknesses. Onboarding needs to be supported by top management, supervisors, colleagues and employees. Professional socialisation can be promoted by providing sufficient time for new hires to familiarise themselves with the work environment and learn about the work community. Thus, it is essential that front‐line nurse manager onboarding be viewed as a long‐term professional development process, whose success is crucial to the work community and the organisation.

Our findings can inform organisational policies and onboarding programme design by emphasising the need for consistent, up‐to‐date and well‐structured onboarding practices. For example, providing standardised orientation materials and technological support ensures clear and predictable guidance for a front‐line nurse manager. They also highlight the importance of fostering both proactive engagement and structured socialisation. For example, assigning mentors or support persons can help build community and provide regular feedback to support the front‐line nurse manager’s adjustment.

## 6. Conclusions

Our study suggests that effective onboarding of front‐line nurse managers should be implemented systematically and should include personal, organisational and social aspects. Front‐line nurse manager onboarding should focus on developing their leadership qualities. Motivation, commitment and attachment to the front‐line nurse manager role should be considered when planning and executing onboarding. Ensuring a structured, comprehensive onboarding programme that highlights the professional requirements of the front‐line nurse manager and the relevant theoretical and practical aspects of managerial work supports the transition to the new role. Alongside structurality, onboarding should be conducted systematically. This can be facilitated by the fluent use of technologies and personnel during the onboarding process.

Versatile support from the work community is crucial during the onboarding of front‐line nurse managers. Organisational support should also be comprehensive. Front‐line nurse managers’ socialisation occurs throughout the onboarding process and can be improved by familiarising them with the work environment and orienting them to the work community.

## Funding

This review did not receive any external funding. Open‐access publishing was facilitated by Oulun Yliopisto, as part of the Wiley–FinELib agreement.

## Ethics Statement

Permission was obtained from the target organisations before the commencement of the study. According to Finnish legislation [37], approval from the research ethics committee is not required if the research does not involve patients or minors and poses no direct or indirect physical or mental harm to the participants [34]. The research was neither sensitive nor posed any risk to participants’ safety or to the researcher. All participants provided their informed consent before participating in the study.

## Conflicts of Interest

The authors declare no conflicts of interest.

## Supporting Information

Additional supporting information can be found online in the Supporting Information section.

## Supporting information


**Supporting Information 1** Supporting File 1. Statement from the Ethics Committee of Human Sciences, University of Oulu, Finland.

**Supporting Information 2** Supporting Figure 1. Nurse managers’ perceptions on effective onboarding for front‐line nurse managers in healthcare (*n* = 18).

## Data Availability

The data that support the findings of this study are available from the corresponding author upon reasonable request.
