# Supplementary material for: Effective Onboarding for Front‐Line Nurse Managers in Healthcare: A Descriptive Qualitative Study
Source: J Nurs Manag. 2026 Apr 28;2026:9942076. doi: 10.1155/jonm/9942076 (PMC13125340; doi:10.1155/jonm/9942076)
Supplement: Supplementary file 2 — Supporting Information 2 Supporting Figure 1. Nurse managers’ perceptions on effective onboarding for front‐line nurse managers in healthcare (n = 18). [file JONM-2026-9942076-s002.pptx]

## Slide 1
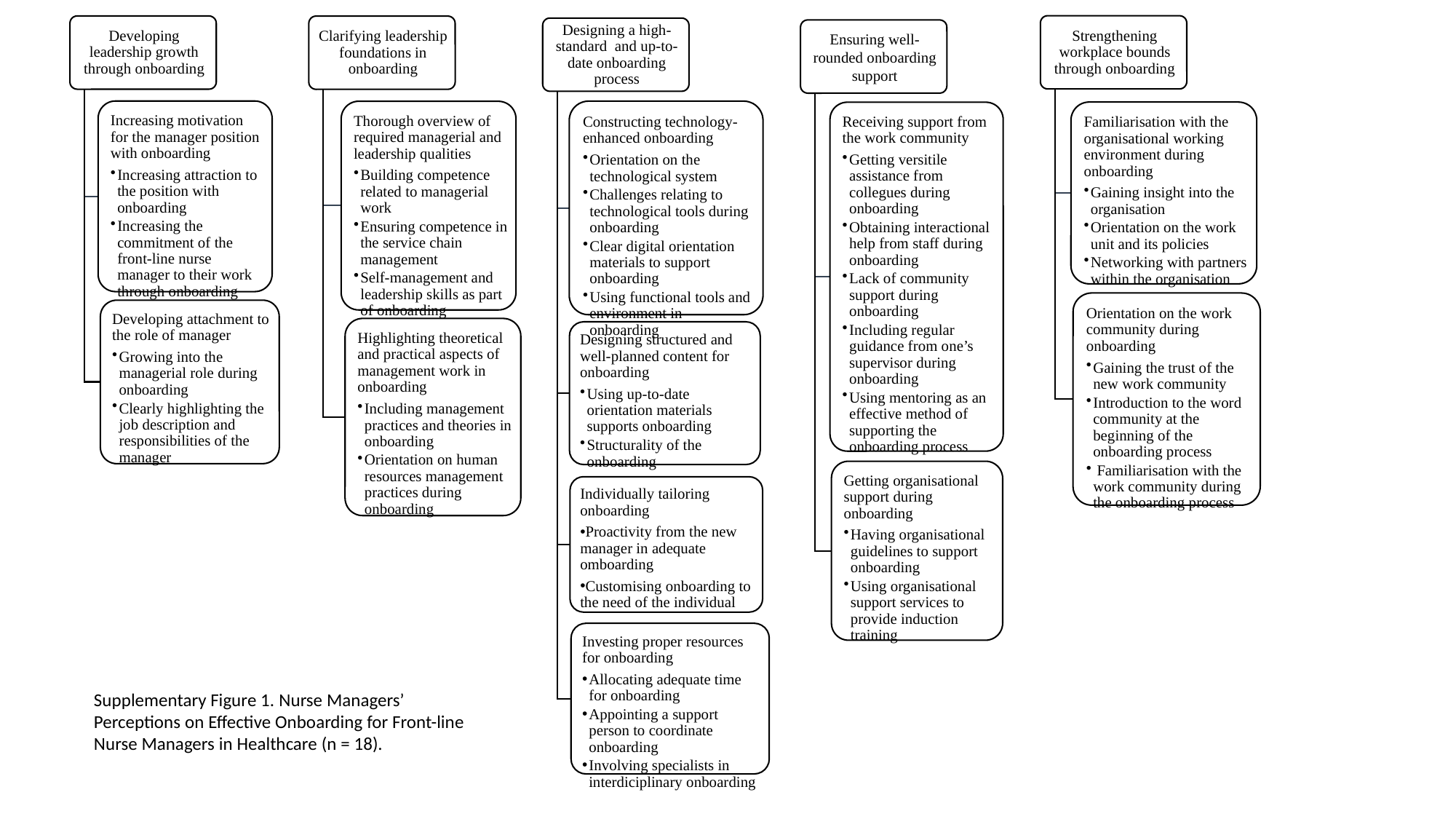

Supplementary Figure 1. Nurse Managers’ Perceptions on Effective Onboarding for Front-line Nurse Managers in Healthcare (n = 18).
